# SARS-CoV-2 (COVID 19) Infection in Hypertensive Patients and in Patients With Cardiac Disease

**DOI:** 10.7759/cureus.8557

**Published:** 2020-06-11

**Authors:** Ivan Cancarevic, Bilal Haider Malik

**Affiliations:** 1 Internal Medicine, California Institute of Behavioral Neurosciences & Psychology, Fairfield, USA; 2 Internal Medicine, California Institute of Behavioral Neurosciences and Psychology, Fairfield, USA

**Keywords:** covid 19, sars-cov-2 (severe acute respiratory syndrome coronavirus -2), novel coronavirus, idiopathic hypertension, heart failure, coronary artery disease, ischemic heart disease, acute respiratory distress syndrome

## Abstract

COVID 19, caused by the SARS-CoV-2 virus, a newly discovered coronavirus, has caused the global pandemic of early 2020. The first case was described in December 2019 in Wuhan, China, and by March 2020, most countries around the world have put in place some of the strictest restrictions seen in decades in order to slow down the spread of the disease. Patients with pre-existing hypertension and cardiovascular comorbidities were reported to be at an increased risk of serious infections caused by SARS-CoV-2. Considering that those are among the most common chronic medical conditions in the Western world, the potential impact of it is huge. The proposed mechanism behind those associations is the expression of angiotensin converting enzyme II (ACE II) in those patients. Furthermore, the association between ACE inhibitors/AR blockers, which are among the most frequently prescribed medications, and serious cases of COVID 19 has been studied with the same mechanism in mind. The reports on the association between hypertension and COVID 19 morbidity and mortality are less clear, and the International Society of Hypertension even claims that there is none. The reports on the association between heart failure or coronary disease and COVID 19 are more uniform, and all seem to point to a greater risk from serious infections faced by patients with those comorbidities. A significant effort will need to be invested by the scientific community into finding strategies for protecting those patients from contracting the virus in the first place and then, once infected, into developing management plans aimed at preserving cardiac function as much as possible.

## Introduction and background

The SARS-CoV-2 virus, a newly discovered member of the coronavirus family, was first described at the end of 2019 in Wuhan, China, as the third severe respiratory syndrome caused by a coronavirus [1]. Due to its easy spread via droplets and direct contact, it quickly spread globally, leading the World Health Organization to declare it a public health emergency [2]. Most countries around the world are taking measures aimed at preventing the spread of the coronavirus as the main mechanism of fighting the disease [2]. Some of those measures have had a significant impact on people's lives. Many countries have imposed travel restrictions [3-4], leaving millions of people having to change travel plans. The psychological and economic impact of mandatory isolation following possible exposure to the virus is another matter of concern [5-7].

Clinically, the disease presents after an incubation period of between 2 and 14 days [8]. The CDC reports that the most common symptoms are fever, cough, and shortness of breath, while dyspnea, chest pain, confusion, and cyanosis represent alarm symptoms that should prompt people to seek medical attention [8]. The largest meta-analysis available at the time of writing this article reports that fever and cough are the presenting symptoms in 89% and 72% of patients, respectively [9]. Fatigue and gastrointestinal symptoms were also frequently reported [10]. Hypertension and diabetes mellitus were the most commonly reported comorbidities [10-12]. Older individuals and those with comorbidities were more likely to develop end-organ damage requiring ICU admission [11]. Acute respiratory distress syndrome (ARDS), acute kidney injury (AKI), and cardiac injury were all reported in severe cases, with ARDS being the most common [11]. Leukopenia and, less commonly, eosinopenia were the most typical laboratory findings, while bilateral ground-glass or patchy opacities in the lungs were the most common radiologic abnormalities [10-12]. C-reactive protein, D-dimer, and procalcitonin levels were elevated, especially in cases of severe disease [12]. At the time of this writing, no clear guidelines on treating SARS-CoV-2 infections have been published, and most clinicians seem to be treating them in a manner similar to that of seasonal flu.

The purpose of this article is to look at the impact the SARS-CoV-2 infection is having on hypertensive patients. Considering the extremely high prevalence of hypertension in society and the fact that hypertension is among the most frequent comorbidities of patients with severe cases of SARS-CoV-2 infections, we believe it is important to look more deeply into the relationship between the two conditions.

## Review

The role of angiotensin converting enzyme

The angiotensin converting enzyme (ACE) enzyme is an essential component of human metabolism, and its dysregulation is associated with a number of different clinical conditions, including hypertension. Many studies were done to assess the potential role of ACE in ARDS development. Multiple animal models have shown a protective effect of ACE II against ARDS development [13-15]. SARS-CoV-2, similarly to SARS-CoV, uses the ACE II receptor to invade the alveolar epithelium and can be considered a potential target for future anti-COVID-19 therapies [13, 16]. Cardiovascular pathology and ACE inhibitors/angiotensin receptor (AR) blockers lead to increased ACE II levels, potentially increasing the risk of SARS-CoV-2 infections, as pointed out by Hanff et al. [17]. There is also some evidence that SARS-CoV-2 can lead to the downregulation of ACE II and toxic accumulation of angiotensin II [17]. It should also be noted that ACE II is expressed in other organs and tissues as well. The fact that ACE is expressed in the gut, heart, and kidney and that SARS-CoV infections can be associated with gastrointestinal symptoms (and the virus can often be isolated from the stool), cardiac damage, and AKI speaks in favor of there being a strong correlation between ACE expression and COVID infection [18].

Overall, the exact role of ACE enzyme and angiotensin in lung injury in general, as well as in the pathogenesis of SARS-CoV-2 (COVID 19) infections, is not clear. If the models are true and ACE II does have a protective role against ARDS and ARDS is the feared complication of COVID 19, it would be reasonable to assume ACE II would have a protective effect against COVID 19. As the ACE II receptor is used by the virus to infect the alveolar epithelium, it raises the issue of whether the availability of the receptor is associated with the severity of clinical findings. Moreover, a large percentage of the population is treated with ACE inhibitors and ARBs. Whether those treatments are associated with a change in outcomes of COVID 19 infections is currently debated. While we recognize the difficulties of performing basic science research, especially on animal models, in light of the current epidemic, we agree with Hanff et al. and would strongly encourage scientists to keep researching the possible connection between ACE receptor, angiotensin, and ARDS.

ACE inhibitors/AR blockers

The ACE inhibitors and AR blockers are currently among the most frequently prescribed medications in the Western world. They are most commonly used in patients suffering from hypertension, congestive heart failure, and proteinuria (most commonly due to diabetes mellitus). They act by reducing the activity of the renin-angiotensin system (RAS). If the RAS plays a significant role in SARS-CoV-19 pathogenesis and ARDS development, it would be reasonable to assume that patients treated with ACE inhibitors/AR blockers would be impacted. The animal models suggest that the administration of ACE inhibitors and AR blockers leads to an elevated number of ACE II receptors in the pulmonary circulation [19]. Marin reported that many patients from Latin America and Spain were intending to discontinue taking their ACE inhibitors/AR blockers due to fear of the infection [20]. Despite some animal models showing a potential association between ACE inhibition and worsening lung injuries due to SARS-CoV, there has been no clinical evidence to support such an association [21]. The European Society of Cardiology recommends not discontinuing either of the drugs as the potential risks associated with uncontrolled hypertension seem to outweigh the risks of ACE inhibition worsening the COVID 19 infection [22]. Danser et al. reached the same conclusion [23]. French health minister claimed that the use of anti-inflammatory medications such as ibuprofen could worsen COVID 19 symptoms [24]. Although ACE II levels can be increased by the use of NSAIDs, the evidence for an association with the severity of COVID 19 symptoms is weak [25]. Fang et al. do, however, recommend monitoring patients who are taking any of the controversial drugs and, when possible, choosing alternative treatments [25]. Despite that, most health authorities, including the NHS, suggest using paracetamol/acetaminophen instead as a precautionary measure [26].

The overall impact of ACE inhibition on SARS-CoV-19 (COVID 19) pathogenesis is at this point of time difficult to establish. The belief that an association between the two exists is mostly based on animal models, while the clinical evidence for such association is lacking. Despite the lack of clinical evidence, it appears to be the case that a number of patients plan on discontinuing their long-term therapies believing that this would decrease their risk of contracting and/or dying from COVID 19. The evidence for a relationship between NSAID use and more severe SARS-CoV-19 infections is also weak, although a possible pathophysiologic link can be hypothesized. In this case, however, many health authorities recommend against using NSAIDs until further evidence comes to light. We believe that a lot more basic science research into the pathophysiology of both coronavirus infections and ARDS development, in general, is needed before clearer patterns emerge. At this point, it may be reasonable to avoid starting new patients on NSAIDs and ACE inhibitors/ARBs if it can be avoided. When it comes to patients who require those treatments for other conditions, discontinuation of them is unlikely to benefit patients as the evidence for exacerbations of their comorbidities is stronger than the evidence for the COVID 19 disease severity.

Hypertension and COVID 19

There have been news reports and expert opinions on patients with hypertension presenting with more severe cases of COVID 19. Moreover, hypertension was among the most frequently reported comorbidities of those patients [10-12]. It should be taken into account, however, that hypertension is among the most prevalent chronic conditions in the Western world, affecting millions of people. Fang et al. point out that the association between diabetes and hypertension and COVID 19 infections is likely due to their association with ACE II enzyme [25]. Li et al. reported a significantly higher prevalence of hypertension and other cardiovascular comorbidities among COVID 19 patients treated in the ICU compared to those who did not require ICU treatment [27]. They also reported that over 8% of those infected by SARS-CoV-2 developed cardiac injury [27]. Chen et al. have also reported a similar association [28]. The elevated troponin levels seen in ICU patients also pointed towards the development of myocardial injury [28]. The odds ratio of developing a severe case compared to a nonsevere case for hypertensive patients was 2.36, with a 95% confidence interval between 1.46 and 3.83 [29]. None of the studies analyzed age as a potential confounder considering that the prevalence of hypertension rises with age, and older patients are, in general, more likely to suffer serious complications of infectious diseases. That was addressed by the International Society of Hypertension, who point out that there is no evidence that patients with hypertension are overrepresented among those with severe SARS-CoV-2 infections when adjusted for age [30].

It is difficult to assess the impact hypertension has on the outcome of patients infected by SARS-CoV-2. Multiple studies point to a significant association between hypertension and adverse outcomes; however, the number of potential confounders, the main of which is age is vast. The previously mentioned association between antihypertensives and severe COVID 19 presentations also needs to be taken into account. The number of studies that have been published so far is insufficient to properly address all those questions. We hope that as more case reports and review articles become available in the literature, a clearer pattern will emerge. The outcomes of patients with uncontrolled hypertension should be compared to those of normotensive controls of similar baseline characteristics. The outcomes of hypertensive patients managed with ACE inhibitors/AR blockers should be compared to those of hypertensive patients managed with calcium channel antagonists.

Coronary artery disease, congestive heart failure, and COVID 19

Besides hypertension, other cardiovascular comorbidities were also frequently reported in patients with severe cases of COVID 19. Considering the significant associations between hypertension and cardiac disease, a correlation would be expected. Moreover, there is a significant association between heart failure and pulmonary disease, particularly pulmonary hypertension. The possible consequences of even mild cardiac damage would be expected to be more significant among patients with pre-existing cardiac disease. As Li et al. reported, the cardio-cerebrovascular disease was among the most prevalent comorbidities among patients with severe cases of COVID 19, second only to hypertension (17.1% and 16.4%, respectively) [27]. Zhou et al. reported that coronary disease was the third most prevalent comorbidity, after hypertension and diabetes [31]. Chen et al. also found that previous history of coronary disease was an independent risk factor for adverse outcomes [28]. The proposed mechanism of such findings is that pericytes with high ACE II expression might be the cells targeted by SARS-CoV-2 [32]. Pericyte injury could then lead to endothelial dysfunction [32]. Patients with pre-existing heart failure are more likely to have elevated ACE II expression due to the pathophysiology of CHF, which could then increase the risk of infection [32]. He et al. found the mortality of critically ill patients infected by SARS-CoV-2 who develop cardiac injury to be as high as 60% [33]. They also found an elevation in N-terminal pro-B-type natriuretic peptide levels (usually a biomarker for congestive heart failure) associated with the myocardial injury caused by COVID 19.

Considered together, these studies point to an association between pre-existing cardiovascular comorbidities and adverse outcomes following SARS-CoV-2 infections. The most likely contributing factor to such finding is the observed myocardial damage in a subset of COVID 19 patients which patients with pre-existing heart disease are less likely to recover from. Moreover, the increased expression of ACE II with congestive heart failure could explain the increased susceptibility to the virus. The ability to address myocardial damage in the ICU may prove to be the most important prognostic factor for those patients. Considering the lack of currently approved treatments aimed at the virus itself, it is especially important to ensure that people with such comorbidities are not exposed to it in the first place. More research is needed into the exact impact of SARS-CoV-2 on the cardiovascular system in order to develop both protective and, potentially, curative strategies.

## Conclusions

COVID 19, caused by SARS-CoV-19, has been the most talked-about disease of the first half of 2020. The elderly and those suffering from chronic conditions are most at risk for adverse outcomes. Hypertension was the most frequently reported comorbidity among patients with severe infections, followed by diabetes and other cardiovascular diseases; possibly related to their expression of ACE II facilitating viral entry into cells. The relationship between ACE inhibitor/AR blocker use and the incidence of severe COVID-19 has been conflicting and at present, where most authorities do not recommend discontinuing long-term ACE inhibitor/AR blocker treatments. A concern remains that some patients may be inclined to stop taking the prescribed medicines out of fear. Another point of contention has been the use of NSAIDs and a number of public health authorities have released statements claiming no evidence of such association existed. Nevertheless, most recommended choosing alternative drugs if possible, pending further investigation into NSAIDs. Finally, the available evidence clearly points to a correlation between pre-existing cardiovascular comorbidities and adverse outcomes with SARS-CoV-2 infections as severe cases are associated with myocardial damage. Considering the very high prevalence of hypertension and cardiovascular diseases, it is important to continue researching the association between these conditions and COVID-19.
